# Effect of stearidonic acid-enriched soybean oil on fatty acid profile and metabolic parameters in lean and obese Zucker rats

**DOI:** 10.1186/1476-511X-12-147

**Published:** 2013-10-19

**Authors:** John M Casey, William J Banz, Elaine S Krul, Dustie N Butteiger, Daniel A Goldstein, Jeremy E Davis

**Affiliations:** 1 Department of Animal Science, Food & Nutrition, Southern Illinois University, Carbondale, IL 62901, USA; 2 Solae, LLC, St. Louis, MO 63188, USA; 3 Monsanto Company, St Louis, MO 63167, USA

**Keywords:** Stearidonic acid, Soybean oil, Obesity, Zucker, Fish oil, Flaxseed oil, Lipids, Hepatic steatosis

## Abstract

**Background:**

Consumption of marine-based oils high in omega-3 polyunsaturated fatty acids (n3PUFAs), eicosapentaenoic acid (EPA) and docosahexaenoic acid (DHA) is known to protect against obesity-related pathologies. It is less clear whether traditional vegetable oils with high omega-6 polyunsaturated fatty acid (n6PUFA) content exhibit similar therapeutic benefits. As such, this study examined the metabolic effects of a plant-based n3PUFA, stearidonic acid (SDA), in polygenic obese rodents.

**Methods:**

Lean (LZR) and obese Zucker (OZR) rats were provided either a standard westernized control diet (CON) with a high n6PUFA to n3PUFA ratio (i.e., 16.2/1.0) or experimental diet modified with flaxseed (FLAX), menhaden (FISH), or SDA oil that resulted in n6PUFA to n3PUFA ratios of 1.7/1.0, 1.3/1.0, and 1.0/0.8, respectively.

**Results:**

After 12 weeks, total adiposity, dyslipidemia, glucose intolerance, and hepatic steatosis were all greater, whereas n3PUFA content in liver, adipose, and muscle was lower in OZR vs. LZR rats. Obese rodents fed modified FISH or SDA diets had lower serum lipids and hepatic fat content vs. CON. The omega-3 index (i.e., ΣEPA + DHA in erythrocyte membrane) was 4.0, 2.4, and 2.0-fold greater in rodents provided FISH, SDA, and FLAX vs. CON diet, irrespective of genotype. Total hepatic n3PUFA and DHA was highest in rats fed FISH, whereas both hepatic and extra-hepatic EPA was higher with FISH and SDA groups.

**Conclusions:**

These data indicate that SDA oil represents a viable plant-derived source of n3PUFA, which has therapeutic implications for several obesity-related pathologies.

## Background

Epidemiological and interventional studies [1-3] have shown that dietary intake of omega-3 polyunsaturated fatty acids (n3PUFAs) such as eicosapentaenoic acid (EPA; 20:5 n3) and docosahexaenoic acid (DHA; 22:6 n3) are associated with a reduced risk of metabolic disease. Additional evidence has demonstrated a therapeutic role of n3PUFAs on obesity-related pathologies including inflammation, dyslipidemia, and insulin resistance [4-6]. EPA and DHA consumption is associated with a reduced risk of sudden death and death from coronary artery disease, which forms the basis of the American Heart Association’s recommendation that individuals with documented coronary disease consume about 1.0 g/d of EPA/DHA [7]. It remains to be determined whether the cardioprotective effect of the long chain n3PUFA are due to effects on metabolism in general or due to cardiac specific effects.

Nonalcoholic fatty liver disease (NAFLD), characterized by excessive hepatic fat accumulation, is associated with increased risk of cardiovascular disease [8]. Current treatment modalities for NAFLD are primarily based on weight loss and lifestyle modification [9]. However, scientific evidence in the form of clinical studies is lacking in this area; thus, the relative efficacy of various approaches remains unknown for the majority of the population. On the other hand, EPA and DHA intake is reported to consistently protect against hepatic steatosis [10-12]. In support of this, a recent meta-analysis [13] confirmed that n3PUFA supplementation effectively reduced liver fat in patients diagnosed with NAFLD.

In Europe as well as the United States, dietary intake of EPA and DHA is well below recommended levels [14,15]. Potential reasons for this disparity include food preferences, economic limitations, and concerns regarding environmental contaminants [16,17]. Additional dietary sources of n3PUFAs—such as flaxseed, canola, and soybean—represent an alternative to fish and fish oils. However, plant-based n3PUFAs are typically higher in α-linolenic acid (ALA; 18:3 n3) compared to EPA and DHA [18]. Although ALA can be converted to EPA and DHA, the overall efficiency is low with conversion ranging from 0.01% to 8% in males or up to 21% in females [19,20].

The rate limiting step for biosynthesis of EPA from ALA is catalyzed by *delta-6 desaturase* (*Fads2*). The product of this specific reaction is stearidonic acid (SDA; 18:4 n3), which is readily catalyzed to EPA by the enzymes *elongase* (*Elovl2/5*) and *delta-5 desaturase* (*Fads1*) [21]. SDA concentrations in marine and plant based oils are typically low; however, it can be intentionally increased in legumes, such as soybean through biotechnology [15]. The consumption of SDA-ethyl esters or SDA-enriched soybean oil is shown to enhance EPA enrichment in humans [22-25]. James et al. [23] specifically demonstrated that the relative efficiency of SDA to enhance EPA concentration in erythrocytes was about 16%, whereas ALA was ~7%. Such observations underlie the potential benefit of SDA-enriched soybean oil to increase *in vivo* concentration of long chain n3PUFA.

Currently, there is only a limited amount of data on the relationship between dietary intake of high SDA oils and obesity-associated pathologies. Two studies with echium oil (~12% SDA) have reported anti-hyperlipidemic and hepatoprotective effects in obesity [26,27]. As such, there is a strong likelihood that SDA-enriched soybean oil may have similar impact on the progression of obesity-related comorbidities. The objectives of the present study were to (*i*) characterize the effect of SDA-enriched soybean oil on n3PUFA enrichment and metabolic dysfunction in obese rodents, and (*ii*) compare and contrast these effects with traditional marine (i.e. menhaden oil) and plant-based (i.e., flaxseed oil) sources of n3PUFAs.

## Methods

### Animals and diets

Twenty-four male homozygous OZR (*fa*/*fa*), and age-matched LZR (+/*fa*) rats (Harlan Laboratory, Indianapolis, IN) were randomly assigned to four diet groups (n = 6) at six weeks of age. Animals were fed *ad libitum* while housed in individual hanging wire cages in a temperature controlled room with a 12 hour light–dark cycle. Prior to termination, animals were fasted overnight. All experimental protocols applied for animal care and use were approved by the Animal Care and Use Committee of Southern Illinois University, Carbondale, IL.

All animals were acclimated on a standard rodent chow for one week prior to study initiation. Control (CON) and experimental diets (Research Diets, Inc., New Brunswick, NJ) were modified from the previously used US17 Monsanto diet [28]. All diets were formulated to be isocaloric and isonitrogenous (Table 1). The CON diet was designed to reflect a typical Western diet with a high n6PUFA to n3PUFA ratio (i.e., 16.2 to 1). The n6PUFA and n3PUFA content of experimental diets was modified by incorporation of flaxseed (FLAX), menhaden (FISH), or SDA oil. To ensure that saturated and monounsaturated fat content was consistent in the experimental diets, the percentage of FLAX, FISH, or SDA oil used was varied (i.e., 7.4%, 12.6%, and 20.6% *of total kcal*, respectively). As such, the PUFA to saturated fat ratio was close to 1.0 for all diets. To ensure limited peroxidation of oils, all diets were stored at -20°C and provided daily. The fatty acid analysis for each diet is presented in Table 1.

**Table 1:** **Composition of experimental diets**^**†**^

**Ingredients**^**‡ **^**( *g/kg *)**	**CON**	**FLAX**	**FISH**	**SDA**
Casein, Sodium	200	200	200	200
L-Cystine	3	3	3	3
Corn Starch	240	240	240	240
Maltodextrin	75	75	75	75
Sucrose	100	100	100	100
Cellulose	50	50	50	50
Cocoa Butter, Deodorized	37.5	37.5	37.5	37.5
Coconut Oil	2.5	2.5	2.5	10.6
Flaxseed Oil	4.5	31.5	4.5	4.5
Menhaden Oil	--	--	53	--
Palm Oil, Deodorized	50	50	24	8.8
Safflower Oil	55.5	28.5	28.5	--
SDA Soybean Oil	--	--	--	88.6
Fatty acids composition^*^( *% of total fat *)				
**ΣSFA**	38.82	38.77	38.77	35.39
**ΣMUFA**	28.01	28.29	26.71	22.26
**Σn3PUFA**	1.83	11.93	13.65	23.11
ALA^[18:3]^	1.83	11.93	2.32	9.24
SDA^[18:4]^	--	--	1.01	13.87
EPA^[20:5]^	--	--	4.78	--
DHA^[22:6]^	--	--	4.18	--
**Σn6PUFA**	29.8	20.4	17.78	18.78
LA^[18:2]^	29.8	20.4	17.1	15.1
GLA^[18:3]^	--	--	--	3.68
AA^[20:4]^	--	--	0.32	--

^†^Adapted from US17 Monsanto Diet (21% kcal protein, 44% kcal carbohydrate, 35% kcal fat) [28].

^**‡**^Additional ingredients: t-BHQ, 0.03 g; mineral mix, 10 g; dicalcium phosphate, 13 g; calcium carbonate, 5.5 g; potassium citrate, 16.5 g; vitamin mix, 10 g; choline bitartrate, 2 g; alpha vitamin E acetate (500 IU/g), 0.13 g.

^*^Fatty Acid Analysis provided by NP Analytical Labs (St. Louis, MO).

### Anthropometric and serum measurements

Body weight and food intake were collected daily. Whole body and liver compositions (i.e., lean, fat, and water) were determined using an EchoMRI-900™ Bioanalyzer (Echo Medical Systems, LLC). At 12 weeks, rodents were fasted overnight and euthanized by CO_2_ asphyxiation and decapitation. Trunk blood was collected and used for subsequent analysis. All tissues were snapped frozen in liquid nitrogen prior to storage at -80°C. Extracted serum was analyzed for cholesterol and triacylglycerol (TAG) (Beckman CX4 Chemistry Analyzer, Brea, CA). Additionally, serum insulin (Millipore, Billerica, MA) and glucose (BioVision, Milpitas, CA) were determined using appropriate assays. The fatty acid profile of erythrocyte membranes was also measured using capillary gas chromatography by OmegaQuant, LLC (Sioux Falls, SD) as previously described [22].

### Oral glucose tolerance test (OGTT)

Prior to termination, OGTTs were performed as described [29]. Briefly, a glucose solution (2 g/kg) was administered by oral gavage and blood samples were collected at 0, 15, 30, 60, and 120 min.

### Tissue fatty acid analysis

Liver, brain, adipose tissue (AT), and soleus tissue samples were measured to 500 mg and put into glass test tubes (16×200 mm) with Teflon-lined screw caps, stored at -80°C for 6h, freeze-dried, and then methylated using the NaOCH_3_ and HCl two-step procedure [30]. Methylated fatty acids were then analyzed for fatty acids using a Shimadzu GC-2010 gas chromatograph (Shimadzu Scientific Instruments Inc., Columbia, MD) equipped with a flame ionization detector and a Supelco 100-m SP-2560 fused silica capillary column (0.25 mm i.d. × 0.2 μm film thickness). The helium carrier gas was maintained at a linear velocity of 23 cm/s. The oven temperature was programmed for 135°C for 5 min, then increased at 5°C/min to 165°C, held there for 80 min, then increased at 3°C/min to 180°C, then increased at 5°C/min to 245°C and held there for 9 min. The injector and detector temperatures were set at 255°C. Peaks were identified by comparing the retention times with those of corresponding standards (Nu-Chek Prep, Elysian, MN; Supelco, Bellefonte, PA; and Larodan Fine Chemicals, Malmo, Sweden). Heptadecanoic acid (C17:0) was added to all samples as an internal standard.

### Hepatic transcript abundance

Total RNA was extracted from liver using Tri Reagent (Molecular Research Center, Inc., Cincinnati, OH) and RNeasy mini columns (QIAGEN Inc., Valencia, CA) as previously described [29]. Purified mRNA was reverse transcribed to cDNA with RT2 PCR Array First Strand Kit and assayed with customized RT2 Profiler PCR Arrays (SABiosciences, Frederick, MD) using gene-specific primers (manufacturer’s proprietary primers, sequences not disclosed). cDNA was diluted into RT2 SYBR Green Master Mix (SABiosciences) and quantitative real time PCR was performed using a MyiQ Real-Time PCR Detection System (Bio-Rad, Hercules, CA). Real time PCRs were performed as follows: melting for 10 min at 95 °C, 40 cycles of two-step PCR including melting for 15 sec at 95 °C, annealing for 1 min at 60 °C. All cycle threshold (Ct) values of > 35.0 were considered non-cycling and removed from analysis. The raw data were analyzed with the ^ΔΔ^Ct method [31] using a web-based software program provided by the manufacturer. Data were presented as fold change relative to LZR fed control diet.

### Statistical analysis

Data were tested for normality and analyzed using the mixed-model analysis with Bonferroni adjustment (SAS Institute, Inc., Cary, NC). Diet and genotype were considered fixed effects. The least significant means (LSMEANS) ± standard error of mean (SEM) are presented in tables and figures. When the interaction of main effects was protected by a significant F-value, post hoc comparisons were made using the LSMEANS separation (pdiff) procedure. Differences among LSMEANS were considered significant at P < 0.05. Significant main effects (diet and genotype) are also presented in tables and figure legends. This standard analysis was performed for all measures unless otherwise specified.

## Results

### Body composition and plasma markers

**Figure 1:**
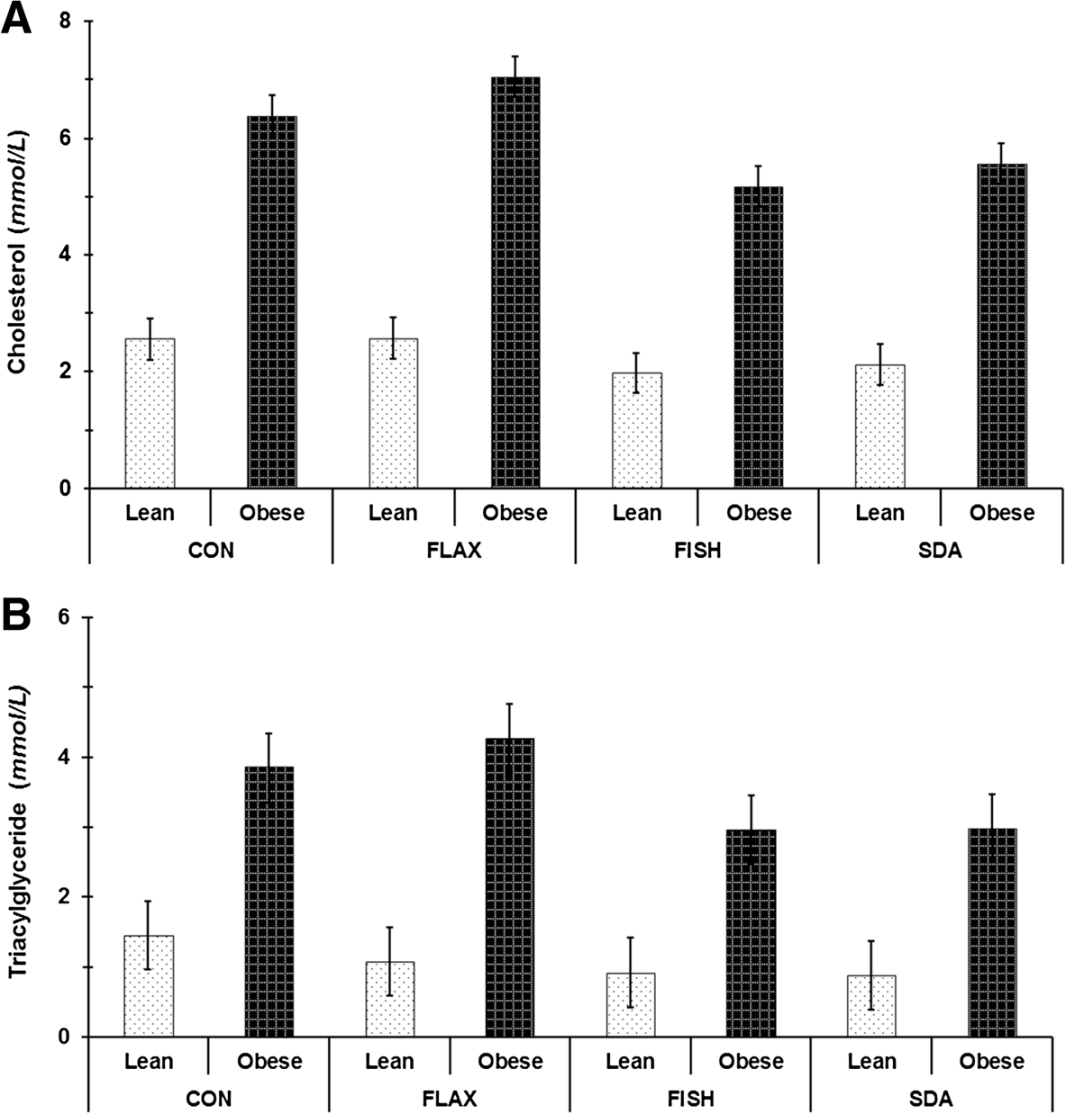
**Serum cholesterol and triacylglyceride concentrations in LZR and OZR rats provided CON, FLAX, FISH, or SDA diets for 12 weeks.** All graphed values represent LS interaction means ± SE. Letters represent significant differences among means as determined by the Bonferroni correction. **A)** Serum cholesterol expressed as mmol/L (n = 6, Genotype effect, P < 0.0001 (Obese > Lean); Diet effect, P < 0.0001 (CON, FLAX > FISH, SDA); Diet*Genotype interaction, P = 0.094). **B)** Serum triacylglyceride expressed as mmol/L (n = 6, Genotype effect, P = 0.0002 (Obese > Lean); Diet effect, P = 0.0065 (CON, FLAX > FISH, SDA); Diet*Genotype interaction, P = 0.93).

Energy intake, body weight, and body fat were greater (Table 2; Genotype, P < 0.0001), whereas lean body mass was lower in obese vs. lean rodents (Genotype, P < 0.0001). There was no significant effect of diet on energy intake (Diet, P = 0.10), body weight (Diet, P = 0.47), body fat mass (Diet, P = 0.07), or lean body mass (Diet, P = 0.61). As expected, dyslipidemia was greater in obese vs. lean rodents (Figure 1; Genotype, P < 0.0001). Serum cholesterol and TAG concentration were lower with SDA or FISH vs. CON and FLAX (Diet, P < 0.0001). Although glucose intolerance, glucose to insulin ratio, and plasma insulin were greater in obese vs. lean rodents (Table 2; Genotype, P = 0.017, P = 0.0003, and P = 0.0057, respectively), there was no significant effect of diet on these variables (Diet, P = 0.35, P = 0.28 and P = 0.25, respectively).

**Table 2:** Morphometric and metabolic parameters in LZR and OZR rats fed CON, FLAX, FISH, or SDA diets for 12 weeks

	**Lean**				**Obese**				**Main effects**		
	**CON**	**FLAX**	**FISH**	**SDA**	**CON**	**FLAX**	**FISH**	**SDA**	**Genotype**	**Diet**	**Interaction**
**Total EI ( *kcal *)**	7588 ± 231	7381 ± 230	7296 ± 228	7276 ± 235	9208 ± 249	9595 ± 219	9069 ± 238	8824 ± 264	P < 0.0001^‡^	P = 0.09	P = 0.30
**Body weight ( *g *)**	497.9 ± 15.7	481.3 ± 15.6	487.8 ± 15.4	485.7 ± 15.9	635.6 ± 16.8	647.7 ± 14.9	626.7 ± 16.1	611.0 ± 17.9	P < 0.0001^‡^	P = 0.47	P = 0.43
**Body fat mass (%)**	15.35 ± 0.98	15.24 ± 0.97	15.53 ± 0.96	13.53 ± 0.99	47.79 ± 1.05	49.86 ± 0.93	48.23 ± 1.01	45.84 ± 1.12	P < 0.0001^‡^	P = 0.0038^i^	P = 0.22
**Body lean mass ( *% *)**	68.27 ± 0.85	68.58 ± 0.84	68.09 ± 0.84	69.68 ± 0.86	40.31 ± 0.91	38.87 ± 0.81	39.75 ± 0.87	41.31 ± 0.97	P < 0.0001^†^	P = 0.050^ii^	P = 0.61
**Adj. liver weight ( *% *)**	2.60 ± 0.16	2.67 ± 0.16	2.88 ± 0.16	2.79 ± 0.16	3.67 ± 0.17	3.75 ± 0.15	3.49 ± 0.16	3.41 ± 0.18	P < 0.0001^‡^	P = 0.81	P = 0.17
**Liver fat mass ( *% *)**	9.15 ± 1.36^*a*^	8.01 ± 1.35^*a*^	10.30 ± 1.34^*a*^	9.22 ± 1.39^*a*^	20.52 ± 1.45^*cd*^	21.72 ± 1.28^*d*^	15.97 ± 1.38^*b*^	18.60 ± 1.48^*bc*^	P < 0.0001^‡^	P = 0.37	^Δ^P = 0.0053
**Liver lean mass (%)**	87.47 ± 1.73^*c*^	85.41 ± 1.72^*c*^	86.10 ± 1.70^*c*^	84.04 ± 1.76^*c*^	73.05 ± 1.44^*a*^	72.94 ± 1.62^*a*^	78.46 ± 1.35^*b*^	76.56 ± 1.48^*b*^	P < 0.0001^†^	P = 0.19	^Δ^P = 0.039
**Glucose (AUC)**	2580 ± 173	2364 ± 171	2618 ± 170	2540 ± 176	2740 ± 184	2544 ± 174	3055 ± 176	3074 ± 189	P = 0.017	P = 0.35	P = 0.46
**Glucose to insulin ratio**	17.69 ± 3.40	23.54 ± 3.32	17.37 ± 3.28	18.46 ± 3.42	1.15 ± 3.50	4.32 ± 3.31	1.21 ± 3.34	1.45 ± 3.59	P = 0.0003^‡^	P = 0.28	P = 0.77
**Insulin ( *pmol/L *)**	0.90 ± 1.10	0.83 ± 1.07	2.32 ± 1.06	1.00 ± 1.10	5.92 ± 1.16	4.09 ± 1.07	6.19 ± 1.08	5.57 ± 1.16	P = 0.0057^‡^	P = 0.25	P = 0.94

^Δ^Different letters represent significance among means as determined by Bonferroni correction (P < 0.05).

Genotype Effect: ^†^Lean > Obese; ^‡^Obese > Lean.

Diet Effect: ^i^CON, FLAX, FISH > SDA; ^ii^SDA > CON, FLAX, FISH.

### Erythrocyte membrane fatty acid composition

**Figure 2:**
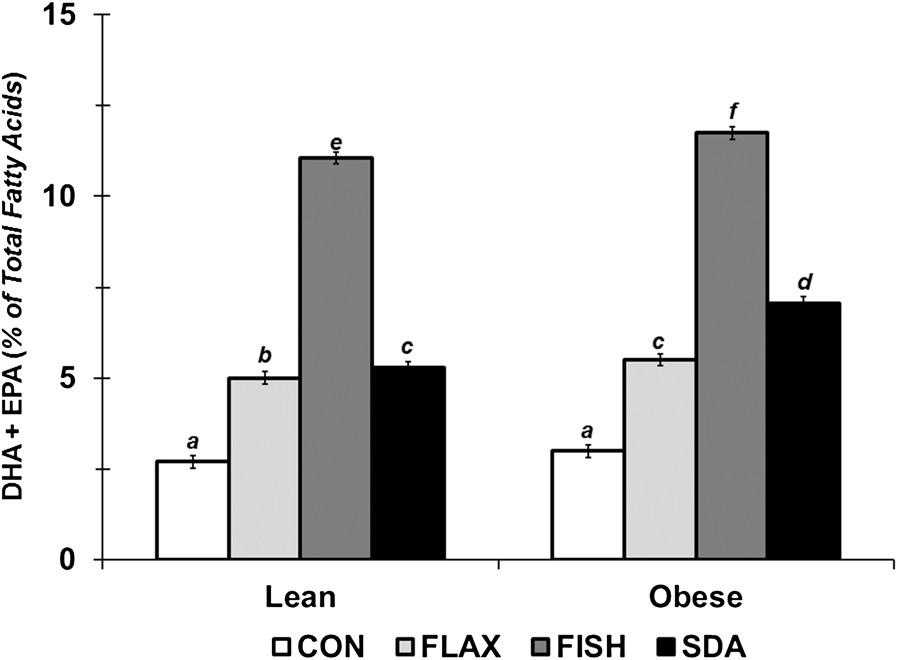
***Erythrocyte membrane Omega-3 Index in LZR and OZR rats fed CON, FLAX, FISH, or SDA diets for 12 weeks*.** Omega-3 Index (%EPA + %DHA) was calculated from RBC membranes as described [22]. All graphed values represent LS interaction means ± SE. Letters represent significant differences among means as determined by the Bonferroni correction (n = 6, Genotype effect, P < 0.0001 (Obese > Lean); Diet effect, P < 0.0001 (FISH > SDA > FLAX > CON); Diet*Genotype interaction, P < 0.0001).

In erythrocytes, the percentage of EPA, DHA, MUFA, and n3PUFA were greater; whereas, LA and n6PUFA were lower in obese vs. lean rodents (Table 3; Genotype, P < 0.001). All n3PUFA-enriched diets (FLAX, FISH, and SDA) increased omega-3 index in OZR rats (Figure 2; Genotype*Diet, P < 0.0001). Moreover, the percentage of EPA, DPA, and n3PUFA were greater; whereas, AA and n6PUFA were lower with FLAX, FISH, or SDA vs. CON (Diet, P < 0.0001). Omega-3 index, as well as the percentage of EPA, DHA, and n3PUFA, was greatest with FISH compared to remaining n3PUFA-enriched diets (Diet, P < 0.0001). In contrast, the percentage of GLA, SDA, and DPA were greatest with SDA (Diet, P < 0.0001). The SDA diet also resulted in a greater percentage of EPA and n3PUFA, as well as a lower percentage of LA, AA, and n6PUFA compared to FLAX (Diet, P < 0.0001).

**Table 3:** Erythrocyte membrane fatty acid composition in LZR and OZR rats fed CON, FLAX, FISH, or SDA diets for 12 weeks

**Fatty acid ( *% of total *)**	**Lean**				**Obese**				**Main effects**		
	**CON**	**FLAX**	**FISH**	**SDA**	**CON**	**FLAX**	**FISH**	**SDA**	**Genotype**	**Diet**	**Interaction**
**LA**^**[18:2(n-6)]**^	9.50 ± 0.28	9.61 ± 0.28	9.72 ± 0.28	8.26 ± 0.29	7.76 ± 0.30	7.97 ± 0.29	8.21 ± 0.29	6.73 ± 0.31	P < 0.0001^†^	P < 0.0001^i^	P = 0.96
**ALA**^**[18:3(n-3)]**^	0.04 ± 0.07^*a*^	0.43 ± 0.07^*b*^	0.10 ± 0.07^*a*^	0.64 ± 0.07^*c*^	0.14 ± 0.07^*a*^	0.70 ± 0.07^*c*^	0.21 ± 0.07^*a*^	0.61 ± 0.08^*bc*^	P = 0.24	P < 0.0001^ii^	^Δ^P = 0.079
**GLA**^**[18:3(n-3)]**^	0.04 ± 0.02	0.04 ± 0.02	0.04 ± 0.02	0.30 ± 0.02	0.05 ± 0.02	0.04 ± 0.02	0.04 ± 0.02	0.20 ± 0.02	P = 0.78	P < 0.0001^iii^	P = 0.52
**SDA**^**[18:4(n-3)]**^	0.03 ± 0.05	0.03 ± 0.05	0.03 ± 0.05	0.40 ± 0.05	0.03 ± 0.03	0.02 ± 0.05	0.03 ± 0.05	0.40 ± 0.05	P = 0.74	P < 0.0001^iii^	P = 0.76
**AA**^**[20:4(n-6)]**^	25.63 ± 0.51	22.21 ± 0.51	15.90 ± 0.50	20.67 ± 0.52	24.91 ± 0.54	21.1 ± 0.51	14.80 ± 0.52	18.13 ± 0.56	P = 0.056	P < 0.0001^iv^	P = 0.13
**EPA**^**[20:5(n-3)]**^	0.05 ± 0.11^*a*^	1.06 ± 0.11^*b*^	5.14 ± 0.11^*f*^	2.80 ± 0.11^*d*^	0.23 ± 0.12^*a*^	1.45 ± 0.11^*c*^	5.41 ± 0.12^*f*^	3.98 ± 0.12^*e*^	P = 0.0017^‡^	P < 0.0001^v^	^Δ^P < 0.0001
**DPA**^**[22:5(n-3)]**^	1.02 ± 0.16^*a*^	3.67 ± 0.16^*cd*^	3.74 ± 0.16^*d*^	5.44 ± 0.17^*e*^	1.19 ± 0.17^*a*^	3.08 ± 0.17^*b*^	3.17 ± 0.18^*bc*^	4.64 ± 0.18^*e*^	P = 0.052	P < 0.0001^vi^	^Δ^P = 0.0050
**DHA**^**[22:6(n-3)]**^	2.51 ± 0.14	3.74 ± 0.14	5.80 ± 0.14	2.31 ± 0.14	2.91 ± 0.15	4.33 ± 0.14	6.50 ± 0.14	3.28 ± 0.15	P = 0.0010^‡^	P < 0.0001^vii^	P = 0.10
**∑SFA**	47.53 ± 0.39	47.58 ± 0.39	47.17 ± 0.38	47.49 ± 0.40	47.35 ± 0.41	47.23 ± 0.39	47.67 ± 0.40	47.31 ± 0.43	P = 0.93	P = 0.99	P = 0.56
**∑MUFA**	9.62 ± 0.39	9.94 ± 0.39	11.00 ± 0.39	10.14 ± 0.40	11.91 ± 0.42	12.12 ± 0.40	12.12 ± 0.40	12.36 ± 0.43	P = 0.0006^‡^	P = 0.11	P = 0.23
**∑n3PUFA**	3.63 ± 0.19	8.92 ± 0.19	14.81 ± 0.19	11.63 ± 0.20	4.48 ± 0.20	9.59 ± 0.19	15.31 ± 0.20	12.88 ± 0.21	P = 0.0029^‡^	P < 0.0001^v^	P = 0.10
**∑n6PUFA**	41.23 ± 0.45	37.23 ± 0.45	32.76 ± 0.44	33.01 ± 0.46	38.61 ± 0.48	35.33 ± 0.45	31.33 ± 0.46	30.67 ± 0.49	P = 0.0013^†^	P < 0.0001^iv^	P = 0.41

^Δ^Different letters represent significance among means as determined by Bonferroni correction (P < 0.05).

Genotype Effect: ^**†**^Lean > Obese; ^**‡**^Obese > Lean.

Diet Effect: ^i^CON, FLAX, FISH > SDA; ^ii^SDA, FLAX > CON, FISH; ^iii^SDA > CON, FLAX, FISH; ^iv^CON > FLAX > SDA > FISH; ^v^FISH > SDA > FLAX > CON; ^vi^SDA > FLAX, FISH > CON; ^vii^FISH > FLAX > SDA, CON.

### Hepatic metabolic profile

Liver weight and fat content were greater; while, lean mass was lower in obese vs. lean rodents (Table 2; Genotype, P < 0.0001). Independently of genotype, there was no significant difference in liver weight (Diet, P = 0.81), fat content (Diet, P = 0.37) or lean mass (Diet, P = 0.19). However, hepatic fat content in obese rodents was unexpectedly greater with FLAX vs. FISH or SDA (Gen*Diet, P < 0.0001). In contrast, the hepatic lean mass in OZR rats was lower with CON and FLAX compared to SDA or FISH (Gen*Diet, P < 0.05).

### Hepatic fatty acid composition

The percentage of ALA, AA, DPA, DHA, MUFA, n3PUFA, and n6PUFA were lower, whereas SFA was greater in liver of obese vs. lean rodents (Table 4; Genotype, P < 0.05). All n3PUFA-enriched diets had a greater percentage of DPA, DHA, and n3PUFA, as well as lower percentage of AA and n6PUFA in liver (Diet, P < 0.0001). The percentage of DPA, DHA and n3PUFA were greater; while the percentage of AA and n6PUFA were lower with FISH vs. FLAX or SDA (Diet, P < 0.05). Alternatively, the percentage of SDA, EPA, DPA, and n3PUFA were greater; whereas, the percentage SFA and MUFA were lower with SDA vs. FLAX (Diet, P < 0.05).

**Table 4:** Hepatic fatty acid composition in LZR and OZR rats fed CON, FLAX, FISH, or SDA diets for 12 weeks

**Fatty acid ( *% of total *)**	**Lean**				**Obese**				**Main effects**		
	**CON**	**FLAX**	**FISH**	**SDA**	**CON**	**FLAX**	**FISH**	**SDA**	**Genotype**	**Diet**	**Interaction**
**LA**^**[18:2(n-6)]**^	1.19 ± 1.41	n.d.	n.d.	2.07 ± 1.42	0.86 ± 1.51	0.55 ± 1.19	0.99 ± 1.42	2.08 ± 1.49	P = 0.71	P = 0.25	P = 0.84
**ALA**^**[18:3(n-3)]**^	0.21 ± 0.17^a^	3.08 ± 0.17^d^	0.93 ± 0.18^b^	3.26 ± 0.17^d^	0.43 ± 0.18^ab^	1.78 ± 0.14^c^	0.63 ± 0.17^ab^	1.88 ± 0.18^c^	P = 0.0016^†^	P < 0.0001^i^	^Δ^P < 0.0001
**SDA**^**[18:4(n-3)]**^	n.d.	0.018 ± 0.26	0.81 ± 0.27	1.07 ± 0.25	0.15 ± 0.27	0.14 ± 0.21	0.19 ± 0.25	0.58 ± 0.26	P = 0.51	P = 0.0011^ii^	P = 0.17
**AA**^**[20:4(n-6)]**^	14.09 ± 0.83^c^	11.41 ± 0.85^b^	3.58 ± 0.89^a^	11.03 ± 0.83^b^	5.53 ± 0.89^a^	4.86 ± 0.70^a^	4.24 ± 0.83^a^	5.97 ± 0.88^a^	P < 0.0001^†^	P < 0.0001^iii^	^Δ^P < 0.0001
**EPA**^**[20:5(n-3)]**^	n.d.	0.50 ± 0.61^a^	6.20 ± 0.63^c^	4.94 ± 0.60^c^	0.63 ± 0.63^a^	0.79 ± 0.50^a^	2.37 ± 0.60^b^	2.67 ± 0.63^b^	P = 0.12	P < 0.0001^ii^	^Δ^P < 0.0001
**DPA**^**[22:5(n-3)]**^	0.12 ± 0.22^a^	1.33 ± 0.23^bc^	3.36 ± 0.24^d^	3.94 ± 0.22^e^	0.42 ± 0.24^ab^	0.77 ± 0.19^b^	1.70 ± 0.22^c^	1.88 ± 0.24^c^	P = 0.0006^†^	P < 0.0001^iv^	^Δ^P < 0.0001
**DHA**^**[22:6(n-3)]**^	2.26 ± 0.39^ab^	4.49 ± 0.40^cd^	10.11 ± 0.42^e^	3.28 ± 0.39^bf^	1.87 ± 0.42^a^	2.43 ± 0.33^ab^	5.63 ± 0.39^d^	3.36 ± 0.41^bc^	P = 0.0007^†^	P < 0.0001^v^	^Δ^P < 0.0001
**∑SFA**	36.48 ± 1.55^c^	36.12 ± 1.59^c^	27.83 ± 1.66^a^	32.41 ± 1.56^b^	38.85 ± 1.66^d^	41.14 ± 1.30^d^	39.88 ± 1.56^d^	39.43 ± 1.64^d^	P = 0.0010^‡^	P = 0.0055^vi^	^Δ^P = 0.0079
**∑MUFA**	41.99 ± 1.90	38.02 ± 1.85	33.93 ± 1.94	31.47 ± 1.82	46.57 ± 1.92	44.30 ± 1.51	39.91 ± 1.80	38.08 ± 1.90	P = 0.032^†^	P = 0.0084^vi^	P = 0.90
**∑n3PUFA**	1.92 ± 1.26^a^	9.38 ± 1.29^b^	21.82 ± 1.35^d^	16.88 ± 1.27^c^	3.65 ± 1.35^a^	6.02 ± 1.06^e^	10.76 ± 1.27^b^	10.94 ± 1.33^b^	P = 0.048^†^	P < 0.0001^iv^	^Δ^P = 0.0057
**∑n6PUFA**	15.28 ± 1.70^a^	10.6 ± 1.74^bc^	3.04 ± 1.82^d^	13.1 ± 1.71^ab^	6.39 ± 1.82^de^	5.41 ± 1.43^de^	5.23 ± 1.71^de^	8.05 ± 1.80^ce^	P = 0.0040^†^	P < 0.0001^iii^	^Δ^P < 0.0001

^Δ^Different letters represent significance among means as determined by Bonferroni correction (P < 0.05).

Genotype Effect: ^**†**^Lean > Obese; ^**‡**^Obese > Lean.

Diet Effect: ^i^SDA, FLAX > FISH, CON; ^ii^SDA, FISH > FLAX, CON; ^iii^CON > SDA, FLAX > FISH; ^iv^FISH > SDA > FLAX > CON; ^v^FISH > SDA, FLAX > CON; ^vi^CON, FLAX > FISH, SDA.

n.d.- not detectable.

Corresponding with these changes in fatty acid profile, there were distinct modification to genes involved in fatty acid elongation and desaturation (Table 5). In particular, hepatic transcript abundance of *Scd1*, *Fads1*, *Fads2*, *Elov5*, *Elov6*, and *PPARα* was greater in obese rodents (P < 0.05). Additionally, transcript abundance of *Fads1*, *Fads2*, *Elvol5*, and *Elvol6* in LZR rats was greater with FLAX vs. SDA or FISH (P < 0.05). Similarly, hepatic transcript abundance of *Fads2*, *Elvol5*, and *Elvol6* in obese rodents was greater with FLAX vs. SDA or FISH (P < 0.05).

**Table 5:** Hepatic transcript abundance in LZR and OZR rats fed CON, FLAX, FISH, or SDA diets for 12 weeks

**Gene symbol**	**Gene name**	**Ref. Seq. #**	**Lean**			**Obese**			
			**FLAX**	**FISH**	**SDA**	**CON**	**FLAX**	**FISH**	**SDA**
**Scd1**	Stearoyl coenzyme A desaturase 1	NM_139192	1.58^a^	1.25^a^	0.43^a^	8.80^b^	10.10^b^	1.49^a^	7.27^b^
**Fads1**	Fatty acid desaturase 1 (*Δ5 desaturase*)	NM_053445	4.92^b^	2.14^a^	0.95^a^	3.49^b^	3.58^b^	3.03^ab^	2.43^ab^
**Fads2**	Fatty acid desaturase 2 (*Δ6 desaturase*)	NM_031344	3.44^b^	2.20^a^	1.01^a^	3.86^b^	4.54^c^	3.57^b^	3.62^b^
**Elovl5**	Elongation of very long chain fatty acids 5	NM_134382	4.41^b^	1.81^a^	0.96^a^	2.81^ab^	4.23^b^	2.28^a^	2.12^a^
**Elovl6**	Elongation of very long chain fatty acids 6	NM_134383	1.94^b^	0.75^a^	0.45^a^	17.39^c^	50.01^d^	11.17^c^	22.84^c^
**Acox1**	Acyl coenzyme A oxidase 1	NM_017340	1.54^a^	1.48^a^	2.23^ab^	1.64^a^	2.22^ab^	2.45^b^	2.54^b^
**PPARα**	Peroxisome proliferator activated receptor α	NM_013196	2.38^a^	1.04^a^	1.00^a^	4.14^b^	4.81^b^	4.56^b^	3.94^b^

Data are expressed as fold change relative to LZR rats fed control diet. Letters represent significance difference among treatments groups as determined by comparison of normalized Ct values (target Ct - housekeeping Ct) with paired t test.

### Extrahepatic fatty acid composition

#### Epidydimal AT

The percentage of ALA, SDA, n3PUFA, and MUFA were lower, whereas the percentage of SFA was greater in epidydimal AT of obese vs. lean rodents (Table 6; Genotype, P < 0.0001). There was also a greater percentage of ALA, DPA, and n3PUFA, as well as lower percentage of AA, MUFA, and n6PUFA with all n3PUFA-enriched diets compared to CON (Diet, P < 0.0001). The percentage of EPA, DPA, DHA, and SFA were greater with FISH vs. FLAX or SDA (Diet, P < 0.0001). In contrast, the percentage of SDA was greater; while, the percentage of LA, MUFA, and SFA was lower with SDA vs. FISH and FLAX (Diet, P < 0.0001). The percentage of ALA was greater with FLAX vs. FISH or SDA (Diet, P < 0.0001). Additionally, the percentage of EPA, DPA, DHA, and AA content was lower with FLAX compared to SDA (P < 0.0001).

**Table 6:** Epidydimal adipose tissue fatty acid profile in LZR and OZR rats fed CON, FLAX, FISH, or SDA diets for 12 weeks

**Fatty acid ( *% of total *)**	**Lean**				**Obese**				**Main effect**		
	**CON**	**FLAX**	**FISH**	**SDA**	**CON**	**FLAX**	**FISH**	**SDA**	**Genotype**	**Diet**	**Interaction**
**LA**^**[18:2(n-6)]**^	1.12 ± 0.18	0.87 ± 0.18	0.81 ± 0.18	0.67 ± 0.18	1.33 ± 0.19	1.10 ± 0.17	1.32 ± 0.20	0.86 ± 0.20	P = 0.25	P = 0.025^i^	P = 0.69
**ALA**^**[18:3(n-3)]**^	1.14 ± 0.10^*ab*^	7.88 ± 0.11^*c*^	1.94 ± 0.10^*e*^	7.47 ± 0.11^*d*^	0.79 ± 0.11^*b*^	5.14 ± 0.10^*f*^	1.17 ± 0.12^*a*^	4.71 ± 0.12^*g*^	P < 0.0001^†^	P < 0.0001^ii^	^Δ^P < 0.0001
**SDA**^**[18:4(n-3)]**^	0.05 ± 0.074^*a*^	0.04 ± 0.08^*a*^	0.28 ± 0.073^*c*^	5.06 ± 0.075^*b*^	0.004 ± 0.08^*a*^	0.005 ± 0.07^*a*^	0.14 ± 0.082^*a*^	2.51 ± 0.082^*d*^	P < 0.0001^†^	P < 0.0001^iii^	^Δ^P < 0.0001
**AA**^**[20:4(n-6)]**^	0.43 ± 0.03	0.27 ± 0.035	0.35 ± 0.034	0.43 ± 0.04	0.56 ± 0.037	0.32 ± 0.032	0.34 ± 0.038	0.45 ± 0.04	P = 0.36	P < 0.0001^iv^	P = 0.15
**EPA**^**[20:5(n-3)]**^	0.14 ± 0.09	0.15 ± 0.087	1.21 ± 0.086	0.59 ± 0.089	n.d.	0.07 ± 0.08	0.88 ± 0.10	0.51 ± 0.10	P = 0.16	P < 0.0001^v^	P = 0.30
**DPA**^**[22:5(n-3)]**^	0.12 ± 0.07^*ad*^	0.24 ± 0.076^*ad*^	0.89 ± 0.073^*c*^	0.61 ± 0.076^*b*^	0.04 ± 0.08^*a*^	0.28 ± 0.070^*d*^	1.05 ± 0.083^*c*^	1.00 ± 0.082^*c*^	P = 0.20	P < 0.0001^vi^	^Δ^P = 0.0029
**DHA**^**[22:6(n-3)]**^	0.11 ± 0.10	0.18 ± 0.10	1.69 ± 0.10	0.22 ± 0.10	0.11 ± 0.10	0.33 ± 0.088	1.45 ± 0.10	0.53 ± 0.10	P = 0.82	P < 0.0001^v^	P = 0.52
**∑SFA**	22.68 ± 0.44^*a*^	23.06 ± 0.44^*a*^	25.66 ± 0.43^*c*^	20.59 ± 0.44^*b*^	27.91 ± 0.47^*d*^	27.84 ± 0.41^*d*^	30.43 ± 0.49^*e*^	27.66 ± 0.48^*d*^	P < 0.0001^‡^	P < 0.0001^vii^	^Δ^P < 0.0001
**∑MUFA**	69.12 ± 0.50^*a*^	62.27 ± 0.51^*b*^	56.64 ± 0.50^*d*^	53.59 ± 0.51^*c*^	61.64 ± 0.54^*b*^	56.39 ± 0.48^*d*^	51.00 ± 0.56^*e*^	50.55 ± 0.56^*e*^	P < 0.0001^†^	P < 0.0001^viii^	^Δ^P = 0.0055
**∑n3PUFA**	1.65 ± 0.36^*a*^	8.61 ± 0.37^*d*^	6.08 ± 0.35^*c*^	14.65 ± 0.37^*f*^	0.83 ± 0.39^*a*^	5.78 ± 0.34^*c*^	4.76 ± 0.40^*b*^	9.92 ± 0.40^*e*^	P < 0.0001^†^	P < 0.0001^ix^	^Δ^P < 0.0001
**∑n6PUFA**	1.55 ± 0.17	1.15 ± 0.17	1.16 ± 0.17	1.12 ± 0.18	1.88 ± 0.18	1.42 ± 0.16	1.66 ± 0.19	1.31 ± 0.19	P = 0.17	P = 0.0046^x^	P = 0.76

^Δ^Different letters represent significance among means as determined by Bonferroni correction (P < 0.05).

Genotype Effect: ^**†**^Lean > Obese; ^**‡**^Obese > Lean.

Diet Effect: ^i^CON, FISH, FLAX > SDA; ^ii^FLAX > SDA > FISH > CON; ^iii^SDA > FISH > CON, FLAX; ^iv^CON > SDA > FISH, FLAX; ^v^FISH > SDA > FLAX, CON; ^vi^FISH > SDA > FLAX > CON; ^vii^FISH > FLAX, CON > SDA; ^viii^CON > FLAX > FISH > SDA; ^ix^SDA > FLAX > FISH > CON; ^x^CON > FISH, FLAX, SDA.

n.d.- not detectable.

#### Subcutaneous AT

The percentage of ALA, SDA, MUFA, n3PUFA were lower; whereas, the percentage of DPA, DHA, and SFA were greater in subcutaneous AT of obese vs. lean rodents (Table 7; Genotype, P < 0.0001). The percentage of DPA and MUFA was greater; while the percentage of ALA and n3PUFA were lower in subcutaneous AT with all n3PUFA-enriched diets (Diet, P < 0.0001). Similar to epidydimal AT, the percentage of EPA, DPA, DHA, and SFA were greater with FISH vs. FLAX or SDA (Diet, P < 0.0001). However, the percentage of SDA, AA, and n3PUFA were greater with SDA vs. FISH or FLAX (Diet, P < 0.0001). Compared to FLAX, the percentage of EPA, DPA, and DHA were lower with SDA (Diet, P < 0.0001).

**Table 7:** Subcutaneous adipose tissue fatty acid profile in LZR and OZR rats fed CON, FLAX, FISH, or SDA diets for 12 weeks

**Fatty acid ( *% of total *)**	**Lean**				**Obese**				**Main effect**		
	**CON**	**FLAX**	**FISH**	**SDA**	**CON**	**FLAX**	**FISH**	**SDA**	**Genotype**	**Diet**	**Interaction**
**LA**^**[18:2(n-6)]**^	0.77 ± 0.24	0.45 ± 0.24	0.57 ± 0.24	0.58 ± 0.24	0.76 ± 0.25	1.16 ± 0.20	0.85 ± 0.24	0.66 ± 0.25	P = 0.36	P = 0.81	P = 0.31
**ALA**^**[18:3(n-3)]**^	0.63 ± 0.15^*ab*^	4.72 ± 0.16^*c*^	1.23 ± 0.14^*d*^	5.03 ± 0.15^*c*^	0.49 ± 0.16^*b*^	3.61 ± 0.12^*e*^	0.92 ± 0.14^*ad*^	3.74 ± 0.15^*e*^	P = 0.0002^†^	P < 0.0001^i^	^Δ^P < 0.0001
**SDA**^**[18:4(n-3)]**^	0.12 ± 0.10^*a*^	0.12 ± 0.11^*a*^	0.26 ± 0.10^*a*^	2.66 ± 0.11^*b*^	n.d.	n.d.	0.02 ± 0.11^*a*^	1.50 ± 0.11^*c*^	P = 0.0011^†^	P < 0.0001^ii^	^Δ^P < 0.0001
**AA**^**[20:4(n-6)]**^	0.28 ± 0.020	0.21 ± 0.021	0.29 ± 0.020	0.36 ± 0.020	0.28 ± 0.021	0.20 ± 0.016	0.25 ± 0.020	0.33 ± 0.021	P = 0.46	P < 0.0001^iii^	P = 0.68
**EPA**^**[20:5(n-3)]**^	0.06 ± 0.03^*a*^	0.08 ± 0.04^*a*^	0.57 ± 0.03^*cd*^	0.28 ± 0.04^*b*^	0.04 ± 0.04^*a*^	0.08 ± 0.03^*a*^	0.62 ± 0.03^*d*^	0.52 ± 0.04^*c*^	P = 0.10	P < 0.0001^v^	^Δ^P < 0.0001
**DPA**^**[22:5(n-3)]**^	0.04 ± 0.03^*a*^	0.01 ± 0.03^*bc*^	0.46 ± 0.03^*e*^	0.32 ± 0.03^*d*^	0.05 ± 0.03^*ab*^	0.16 ± 0.02^*c*^	0.68 ± 0.029^*f*^	0.66 ± 0.031^*f*^	P < 0.0001^‡^	P < 0.0001^vi^	^Δ^P < 0.0001
**DHA**^**[22:6(n-3)]**^	n.d.	0.03 ± 0.04^*ab*^	0.73 ± 0.04^*c*^	0.07 ± 0.04^*ab*^	0.06 ± 0.04^*ab*^	0.13 ± 0.033^*b*^	1.12 ± 0.04^*e*^	0.33 ± 0.041^*d*^	P < 0.0001^‡^	P < 0.0001^v^	^Δ^P < 0.0001
**∑SFA**	26.20 ± 1.06	25.55 ± 1.08	27.46 ± 1.05	24.22 ± 1.09	32.27 ± 1.12	31.20 ± 0.88	34.23 ± 1.05	30.42 ± 1.11	P < 0.0001^‡^	P = 0.0017^vii^	P = 0.94
**∑MUFA**	66.50 ± 1.10	63.54 ± 1.12	61.57 ± 1.09	58.74 ± 1.13	62.66 ± 1.16	60.48 ± 0.91	55.97 ± 1.09	56.71 ± 1.15	P = 0.0083^†^	P < 0.0001^viii^	P = 0.27
**∑n3PUFA**	0.87 ± 0.29^*a*^	5.10 ± 0.30^*c*^	3.42 ± 0.29^*b*^	8.68 ± 0.30^*e*^	0.57 ± 0.31^*a*^	3.98 ± 0.24^*b*^	3.52 ± 0.29^*b*^	7.50 ± 0.31^*d*^	P = 0.084^†^	P < 0.0001^ix^	^Δ^P = 0.030
**∑n6PUFA**	1.05 ± 0.24	0.66 ± 0.24	0.86 ± 0.24	0.94 ± 0.25	1.04 ± 0.25	1.35 ± 0.20	1.10 ± 0.24	1.00 ± 0.25	P = 0.40	P = 0.98	P = 0.32

^Δ^Different letters represent significance among means as determined by Bonferroni correction (P < 0.05).

Genotype Effect: ^**†**^Lean > Obese; ^**‡**^Obese > Lean.

Diet Effect: ^i^SDA, FLAX > FISH > CON; ^ii^SDA > FISH, FLAX, CON; ^iii^SDA > FISH, CON > FLAX; ^iv^SDA > FISH > FLAX, CON; ^v^FISH > SDA > FLAX, CON; ^vi^FISH > SDA > FLAX > CON; ^vii^FISH > CON, FLAX, SDA; ^viii^CON > FLAX > FISH, SDA; ^ix^SDA > FLAX > FISH > CON.

n.d.- not detectable.

#### Soleus muscle

The percentage of SDA and n3PUFA were lower in soleus muscle of obese vs. lean rodents (Table 8; Genotype, P < 0.0001). The percentage of n3PUFA was greater; while, the percentage of AA and n6PUFA were lower with all n3PUFA-enriched diets (Diet, P < 0.0001). Unlike AT depots, there was no difference in EPA or DHA content between FISH and SDA (Diet, P = 0.94 and P = 0.34, respectively). Moreover, the percentage of EPA and DHA was greater with FISH or SDA vs. CON and FLAX (Diet, P < 0.05). The percentage of LA (Diet, P < 0.05), SDA (Diet, P < 0.0001), DPA (Diet, P < 0.0001) and n3PUFA (Diet, P < 0.05) were also greater with SDA vs. FISH or FLAX. Additionally, the percentage of EPA and DPA were greater; whereas, the percentage of MUFA was lower in SDA vs. FLAX (Diet, P < 0.0001).

**Table 8:** Soleus muscle fatty acid profile in LZR and OZR rats fed CON, FLAX, FISH, or SDA diets for 12 weeks

**Fatty acid ( *% of total *)**	**Lean**				**Obese**				**Main effect**		
	**CON**	**FLAX**	**FISH**	**SDA**	**CON**	**FLAX**	**FISH**	**SDA**	**Genotype**	**Diet**	**Interaction**
**LA**^**[18:2(n-6)]**^	0.06 ± 0.02^*ac*^	0.007 ± 0.02^*b*^	0.02 ± 0.02^*abc*^	0.04 ± 0.02^*abc*^	0.03 ± 0.01^*ab*^	0.02 ± 0.01^*ab*^	0.03 ± 0.02^*ab*^	0.07 ± 0.01^*c*^	P = 0.95	P = 0.0045^i^	P = 0.64
**ALA**^**[18:3(n-3)]**^	1.71 ± 0.64	4.79 ± 0.69	1.88 ± 0.67	4.08 ± 0.65	1.80 ± 0.69	3.51 ± 0.54	1.32 ± 0.66	1.80 ± 0.67	P = 0.23	P < 0.0001^ii^	P = 0.18
**SDA**^**[18:4(n-3)]**^	0.66 ± 0.35	0.39 ± 0.38	0.47 ± 0.38	2.72 ± 0.36	0.044 ± 0.38	0.15 ± 0.30	0.19 ± 0.37	0.41 ± 0.37	P = 0.067^†^	P < 0.0001^i^	^Δ^P = 0.0020
**AA**^**[20:4(n-6)]**^	5.37 ± 0.64^*b*^	3.40 ± 0.70^*a*^	1.90 ± 0.68^*a*^	3.58 ± 0.65^*a*^	3.60 ± 0.70^*ab*^	2.90 ± 0.54^*a*^	3.74 ± 0.67^*ab*^	3.63 ± 0.67^*ab*^	P = 0.91	P = 0.026^iii^	^Δ^P = 0.019
**EPA**^**[20:5(n-3)]**^	0.41 ± 0.21	0.48 ± 0.22	1.66 ± 0.22	1.41 ± 0.21	0.32 ± 0.22	0.22 ± 0.17	0.85 ± 0.21	1.11 ± 0.21	P = 0.15	P < 0.0001^iv^	P = 0.18
**DPA**^**[22:5(n-3)]**^	1.19 ± 0.33	1.71 ± 0.36	1.69 ± 0.35	2.87 ± 0.34	0.99 ± 0.36	0.95 ± 0.28	1.51 ± 0.34	2.15 ± 0.35	P = 0.28	P < 0.0001^i^	P = 0.58
**DHA**^**[22:6(n-3)]**^	4.14 ± 1.00^*ac*^	4.82 ± 1.08^*ab*^	7.09 ± 1.06^*b*^	4.06 ± 1.01^*a*^	2.27 ± 1.08^*a*^	1.87 ± 0.84^*a*^	3.57 ± 1.03^*ac*^	5.01 ± 1.04^*c*^	P = 0.16	P = 0.054^iv^	^Δ^P = 0.043
**∑SFA**	29.69 ± 0.64	29.50 ± 0.70	31.17 ± 0.68	28.54 ± 0.65	30.12 ± 0.70	30.15 ± 0.54	32.38 ± 0.67	30.24 ± 0.68	P = 0.24	P = 0.0004^v^	P = 0.65
**∑MUFA**	50.76 ± 2.26	51.57 ± 2.45	46.00 ± 2.39	46.86 ± 2.29	56.05 ± 2.45	50.83 ± 1.90	47.21 ± 2.34	42.56 ± 2.36	P = 0.90	P < 0.0001^vi^	P = 0.093
**∑n3PUFA**	8.32 ± 1.52	12.29 ± 1.65	13.00 ± 1.61	15.63 ± 1.54	5.54 ± 1.65	6.79 ± 1.28	7.67 ± 1.57	10.88 ± 1.59	P = 0.023^†^	P < 0.0001^vii^	P = 0.72
**∑n6PUFA**	5.41 ± 0.65^*c*^	3.41 ± 0.70^*b*^	1.92 ± 0.69^*a*^	3.62 ± 0.66^*b*^	3.62 ± 0.70^*b*^	2.91 ± 0.55^*ab*^	3.76 ± 0.67^*b*^	3.68 ± 0.68^*b*^	P = 0.91	P = 0.026^iii^	^Δ^P = 0.020

^Δ^Different letters represent significance among means as determined by Bonferroni correction (P < 0.05).

Genotype Effect: ^**†**^Lean > Obese; Obese > Lean.

Diet Effect: ^i^SDA > CON, FISH > FLAX; ^ii^FLAX > SDA > FISH, CON; ^iii^CON > SDA, FLAX, FISH; ^iv^SDA, FISH > FLAX, CON; ^v^FISH > CON, FLAX, SDA; ^vi^CON, FLAX > FISH, SDA; ^vii^SDA > FISH, FLAX > CON.

#### Brain

The percentage of EPA, DPA, and DHA were greater, whereas, the percentage of ALA and MUFA were lower in brain tissue of obese vs. lean rodents (Table 9; Genotype, P < 0.0001). Overall, there was a greater percentage of EPA, DHA, and n3PUFA (Diet, P < 0.0001); whereas, the percentage of AA and n6PUFA was lower with all n3PUFA-enriched diets (Diet, P < 0.05). The percentage of EPA and DHA were greater; while, the percentage of AA and n6PUFA was lower with FISH vs. FLAX or SDA (Diet, P < 0.0001). In contrast, the percentage of DPA was greater; while, the percentage of ALA was lower with SDA vs. FISH and FLAX (Diet, P < 0.0001). The percentage of ALA was greater with FLAX vs. FISH or SDA (Diet, P < 0.05). However, the percentage of EPA and n3PUFA was lower with FLAX vs. SDA (Diet, P < 0.0001).

**Table 9:** Brain fatty acid profile in LZR and OZR rats fed CON, FLAX, FISH, or SDA diets for 12 week

**Fatty acid ( *% of total *)**	**Lean**				**Obese**				**Main effect**		
	**CON**	**FLAX**	**FISH**	**SDA**	**CON**	**FLAX**	**FISH**	**SDA**	**Genotype**	**Diet**	**Interaction**
**LA**^**[18:2(n-6)]**^	0.005 ± 0.002	0.009 ± 0.002	0.005 ± 0.002	0.009 ± 0.002	0.007 ± 0.002	0.007 ± 0.001	0.010 ± 0.002	0.012 ± 0.002	P = 0.13	P = 0.48	P = 0.35
**ALA**^**[18:3(n-3)]**^	1.31 ± 0.030	1.36 ± 0.030	1.28 ± 0.028	1.23 ± 0.030	1.19 ± 0.030	1.20 ± 0.024	1.16 ± 0.030	1.05 ± 0.030	P < 0.0001^†^	P < 0.0001^i^	P = 0.58
**SDA**^**[18:4(n-3)]**^	0.16 ± 0.027	0.14 ± 0.027	0.18 ± 0.026	0.18 ± 0.027	0.08 ± 0.028	0.10 ± 0.022	0.13 ± 0.027	0.12 ± 0.027	P = 0.078	P = 0.30	P = 0.83
**AA**^**[20:4(n-6)]**^	11.02 ± 0.20^*de*^	10.69 ± 0.20^*cd*^	9.80 ± 0.19^*b*^	10.65 ± 0.20^*cd*^	11.19 ± 0.21^*e*^	10.27 ± 0.16^*bc*^	8.87 ± 0.20^*a*^	10.48 ± 0.20^*cd*^	P = 0.16	P < 0.0001^ii^	^Δ^P = 0.021
**EPA**^**[20:5(n-3)]**^	0.023 ± 0.01^*a*^	0.028 ± 0.01^*a*^	0.13 ± 0.01^*c*^	0.079 ± 0.01^*b*^	0.018 ± 0.01^*a*^	0.066 ± 0.0^*b*^	0.24 ± 0.01^*e*^	0.20 ± 0.01^*d*^	P < 0.0001^‡^	P < 0.0001^iii^	^Δ^P < 0.0001
**DPA**^**[22:5(n-3)]**^	0.13 ± 0.018^*a*^	0.28 ± 0.018^*b*^	0.46 ± 0.017^*c*^	0.66 ± 0.018^*e*^	0.13 ± 0.018^*a*^	0.33 ± 0.014^*b*^	0.56 ± 0.018^*d*^	0.81 ± 0.018^*f*^	P = 0.0009^‡^	P < 0.0001^iv^	^Δ^P < 0.0001
**DHA**^**[22:6(n-3)]**^	12.80 ± 0.22	13.53 ± 0.21	14.38 ± 0.21	13.53 ± 0.22	13.52 ± 0.22	13.61 ± 0.17	15.06 ± 0.22	13.90 ± 0.22	P = 0.077^‡^	P < 0.0001^v^	P = 0.26
**∑SFA**	36.00 ± 0.27	35.78 ± 0.26	35.74 ± 0.25	35.73 ± 0.26	36.35 ± 0.27	36.06 ± 0.21	35.77 ± 0.26	36.83 ± 0.27	P = 0.16	P = 0.074^vi^	P = 0.091
**∑MUFA**	19.90 ± 0.16	20.44 ± 0.15	20.84 ± 0.15	20.1 ± 0.15	19.61 ± 0.16	20.08 ± 0.12	20.03 ± 0.15	19.82 ± 0.16	P = 0.021^†^	P < 0.0001^vii^	P = 0.14
**∑n3PUFA**	14.62 ± 0.21	15.53 ± 0.20	16.61 ± 0.19	15.85 ± 0.20	15.08 ± 0.21	15.46 ± 0.16	17.23 ± 0.20	16.29 ± 0.21	P = 0.14	P < 0.0001^iii^	P = 0.21
**∑n6PUFA**	11.02 ± 0.20^*bd*^	10.70 ± 0.20^*b*^	9.80 ± 0.19^*b*^	10.65 ± 0.20^*bc*^	11.20 ± 0.21^*d*^	10.28 ± 0.16^*c*^	8.88 ± 0.20^*a*^	10.49 ± 0.20^*b*^	P = 0.17	P < 0.0001^ii^	^Δ^P = 0.023

^Δ^Different letters represent significance among means as determined by Bonferroni correction (P < 0.05).

Genotype Effect: ^**†**^Lean > Obese; ^**‡**^Obese > Lean.

Diet Effect: ^i^FLAX > CON > FISH > SDA; ^ii^CON > FLAX, SDA > FISH; ^iii^FISH > SDA > FLAX > CON; ^iv^SDA > FISH > FLAX > CON; ^v^FISH > SDA, FLAX > CON; ^vi^SDA, CON > FISH, FLAX; ^vii^FISH, FLAX > SDA, CON.

## Discussion

In this study, we examined how incorporation of n3PUFA-enriched soybean oil (SDA) into a westernized diet influenced fatty acid composition and obesity-related comorbidities. Our data show that SDA enhanced n3PUFA profiles in lean and obese rodents. Furthermore, the magnitude of this effect was consistent with established dietary sources of n3PUFA, including marine and plant based oils. The severity of obesity-associated dysfunction was also improved with inclusion of SDA, which was evident from a reduction in serum lipids and ectopic fatty liver. Collectively, these findings show that SDA oil is a viable source of n3PUFAs with potential therapeutic properties consistent with FISH and FLAX [1-3].

As expected, the hyperphagic obese rodents had significantly greater adiposity, dyslipidemia, and glucose intolerance compared to lean counterparts. The omega-3 index was also modestly elevated in OZR rats, which further corresponded to greater induction of PUFA-associated genes. Interestingly, obese rodents exhibited lower hepatic n6PUFA, with a particularly large reduction in AA. This finding was not consistent in extra-hepatic tissues in which n6PUFA concentrations were similar in OZR and LZR rats. Cao et al. [32] similarly showed that fatty acid profiles between lean and obese rodents were most different in the liver, but not in other tissues when animals were provided equal amounts of dietary n3PUFAs (i.e., the hyperphagic OZR rats were pair-fed to lean counterparts). In the current study, diets were fed *ad libitum*, which may account for the observation that AA was relatively lower in obese rats in the current study compared to LA in the study by Cao et al. [32]. As such, the relatively higher intake of n3PUFAs in OZR rats may have specifically competed for the hepatic *desaturases* and *elongases* resulting in lower AA.

The incorporation of dietary n3PUFA had no effect on glucose homeostasis in the polygenic OZR rat. However, previous studies have shown improved insulin sensitivity with increased dietary n3PUFA intake in obese and diabetic mice [6,33]. Such a disparity in results likely reflects differences in the animal models *per se*, use of purified long chain PUFAs versus fish oil, disparities in absolute daily intake of individual fatty acids, and variations in biomarkers used to assess glucose metabolism (i.e., fasting blood glucose vs. oral glucose tolerance). Nonetheless, experimental diets high in long-chain n3PUFA (i.e., FISH or SDA) was associated with reduced fatty liver in obese rats, a finding consistent with studies cited by Fedor et al. [33]. Consequently, these data indicate that hepatic insulin sensitivity may be better maintained with increased consumption of long-chain n3PUFAs.

All experimental diets resulted in greater total n3PUFA and lower n6PUFA enrichment of erythrocytes and liver compared to control (CON). However, the incorporation of a marine-based source of n3PUFA (FISH) had the greatest impact on EPA and DHA enrichment. This effect was consistent in erythrocytes and in the majority of analyzed tissues (excluding skeletal muscle where SDA tended to increase EPA and DHA to a higher degree in obese rats). Previous studies [34,35] have consistently shown fish oil consumption to be the most efficient dietary intervention for increasing overall tissue long chain n3PUFA content. This is undoubtedly due to the large concentration of endogenous EPA and DHA in fish oil, which enriches tissue without the need for additional enzymatic modification *in vivo* as is the case for ALA and to a lesser extent SDA. The differential mRNA abundance of hepatic *desaturase* and *elongase* genes observed in both lean and obese rodents provided FISH or SDA compared to FLAX is consistent with the observation that dietary long-chain PUFAs do down-regulate *Fads1* and *Fads2 in vivo* and *in vitro*[36]. As expected, we also showed the lowest n6PUFA and AA concentrations in erythrocytes, liver, and brain after FISH consumption compared to the other diets. Consumption of SDA resulted in the next lowest n6PUFA and AA concentrations in erythrocytes, while reductions of n6PUFA and AA compared to CON in brain and liver by FLAX and SDA were similar. The reductions in n6PUFAs and AA are likely due to the high endogenous n3PUFA content in fish, SDA-enriched soybean and flaxseed oils, as n3PUFAs have been shown to directly impact the metabolism of n6PUFAs [37].

Despite a lower magnitude of n3PUFA tissue enrichment, the metabolic profile with SDA was comparable to the marine-based oil diet. In particular, we observed similar protection against dyslipidemia and hepatic steatosis with SDA and FISH. These hypolipidemic effects may be attributed to an equivalent rise in hepatic EPA content. Willumsen et al. [38] previously showed that greater hepatic EPA, but not DHA, improved lipid homeostasis through inhibition of VLDL production in rats. Additionally, the high rate of peroxisomal retroconversion of DHA [39] and docosapentaenoic acid (DPA; 22:5 n3) [40] to EPA in rat liver further suggests that EPA may play a more important role in lipid lowering. In our study, the relatively low hepatic DHA content along with marginal SDA levels indicates that the beneficial hypolipidemic properties of SDA are likely related to the increase in EPA biosynthesis following SDA consumption.

Plant-based sources of n3PUFA, such as flaxseed oil, are primarily high in ALA, which exhibits a relatively low *in vivo* conversion to EPA [18]. On the other hand, n3PUFA-enriched soybean oil is high in ALA and SDA. The latter is efficiently converted to EPA as the reaction is not dependent on *delta-6-desaturase* (*Fads2*) activity—the rate limiting enzyme in ALA’s conversion to EPA [22-25]. Accordingly, our data show that the EPA content in erythrocytes, liver, brain, adipose tissue and skeletal muscle was greater with SDA vs. FLAX. This further corresponded with greater total n3PUFA and omega-3 index with SDA compared to FLAX groups. Although it is possible that the lower percentage of flaxseed oil (relative to SDA oil) is responsible for these differences, it has been reported that an increase in dietary ALA from 0.4% to 1.1% (*of total kcal*) reduced ALA conversion from 9% to 3% [41]. In our study, ALA represented 4.2% and 3.0% (*of total kcal*) for FLAX and SDA diets. Thus, incorporation of more flaxseed oil would likely result in less EPA, whereas SDA conversion to EPA would be unaffected by increased ALA.

The lower EPA content in FLAX fed rodents may also be due to greater competition between other fatty acids in the flaxseed oil. For instance, linoleic acid (LA; 18:2 n-6) and oleic acid (OA; 18:1 n-9), are potential substrates for *Fads2* that can also compete with ALA for binding [42]. The increased concentration of these alternate substrates in flaxseed oil can subsequently reduce ALA conversion even further [42,43]. In our study, OA and LA represented 28% and 20% of the total fatty acid content in the FLAX diet, which was also approximately 19% and 40% greater than the OA and LA content of the SDA diet, respectively.

Several studies have suggested that the conversion efficiency of ALA is also influenced by total n3PUFA content. Gibson et al. [44] showed that EPA biosynthesis from ALA was reduced when the total n3PUFA in diet was > 3% of total energy. The amount of n3PUFA in FLAX was > 3% of total energy which would therefore be expected to decrease ALA conversion (FLAX had approximately 12% of total energy from n3PUFAs). We also observed the greatest induction of hepatic transcript abundance for *desaturases* and *elongases* with FLAX. Our findings are consistent with data that showed *desaturase* enzyme activities in rat liver were distinctly increased by flaxseed oil compared to fish oil [45].

In contrast, Igarashi and colleagues [46] reported that deprivation of n3PUFA resulted in a significant enhancement of ALA conversion through upregulation of *Fads1*, *Fads2*, *Elovl2*, and *Elovl5* mRNA in liver; however, they also studied n3PUFA “deficient” diets which may account for the apparent discrepancy to our current observations which were not n3PUFA deficient. More recent work [47] has suggested that ALA conversion is more effectively regulated by fatty acid substrate concentrations than changes in the expression of *desaturase* or *elongase* genes, which may explain how FLAX, which had the greatest enzyme abundance also exhibited the lower EPA biosynthesis compared to SDA.

## Conclusion

The results of the present investigation are consistent with previous data showing that SDA-enriched soybean oil markedly enhanced n3PUFA enrichment as evident from erythrocyte and tissue profiles. Furthermore, we demonstrated that SDA and FISH diets protected against several obesity-related pathologies, including dyslipidemia and hepatic steatosis. Although not fully elucidated, we hypothesize that these hypolipidemic properties were partially attributed to hepatic EPA enrichment. Collectively, these data indicate that SDA-enriched soybean oil is a viable plant-based alternative to traditional marine-based n3PUFA. In addition, incorporation of SDA-enriched soybean oil into the food supply, as a more sustainable food ingredient, may increase overall dietary n3PUFA intake which may help reduce the prevalence of obesity-related disease.

## Competing interests

The authors declare that they have no competing interests.

## Authors’ contributions

WJB, ESK, DNB, DAG, and JED designed study. JMC, WJB, and JED conducted the research. JMC and JED analyzed the data and wrote corresponding manuscript. JED had primary responsibility for the final content. All authors read and approved the final manuscript.
